# Crystal structure of Staudtienic acid, a diterpenoid from *Staudtia kamerunensis* Warb. (Myristicaceae)

**DOI:** 10.1107/S2056989024005000

**Published:** 2024-07-19

**Authors:** Jordan Tonga Lembe, Precious Mokgadi Mphahlele, Michael Hermann Kamdem Kengne, Pangaman Jiyane, Marthe Carine Djuidje Fotsing, Charmaine Ardene, Edwin Mpho Mmutlane, Derek Tantoh Ndinteh

**Affiliations:** a Drug Discovery and Smart Molecules Research Laboratory, Department of Chemical, Sciences, University of Johannesburg, PO Box 17011, Doornfontein, Johannesburg 2028, South Africa; b Centre for Natural Product Research (CNPR), Department of Chemical Sciences, University of Johannesburg, Doornfontein, Johannesburg 2028, South Africa; c Research Centre for Synthesis and Catalysis, Department of Chemical Sciences, University of Johannesburg-Kingsway Campus, Auckland Park 2008, South Africa; Universidad de Los Andes Mérida, Venezuela

**Keywords:** crystal structure, *Staudtia kamerunensis*, Staudtienic acid, Myristicaceae, hydrogen bonding

## Abstract

This title compound, C_20_H_26_O_2_, was isolated from the benzene fraction of the stem bark of *Staudtia kamerunensis* Warb. (Myristicaceae) using column chromatography techniques over silica gel. The compound was fully characterized by single-crystal X-ray diffraction, one and two-dimensional NMR spectroscopy, IR and MS spectrometry. The compound has two fused cyclo­hexane rings attached to a benzene ring, with a carb­oxy­lic acid on C-4. This cyclo­hexene ring has a chair conformation while the other adopts a half-chair conformation. The benzene ring is substituted with a propenyl moiety. The structure is characterized by inter­molecular O—H⋯O hydrogen bonds, two C—H⋯O intra­molecular hydrogen bonds and two C—H⋯π inter­actions. The mol­ecular structure confirms previous studies carried out by spectroscopic techniques.

## Abstract 2

This work presents the crystal structure of a natural diterpenoid, Staudtienic acid, isolated from the stem bark of *Staudtia kamerunensis* Warb (Myristicaceae). This work confirmed the results previously obtained from NOE spectroscopy.

## 1. Chemical context

Diterpenoids are secondary metabolites mainly obtained from plant species but that can also originate from marine species (Hanson, 2009 ▸).

The biological importance of diterpenoids is notable. For example, Clerodanes’ diterpenoids are compounds isolated from several plant sources that have been reported to possess very good anti­feedant activity against certain plant worms such as the tobacco cutworm (*Spodoplera litura*) and certain locusts such as the African desert locust (*Schisticerea gregoria*). They are reported to possess anti­viral, anti­tumour and anti­biotic properties, among many others (Merritt & Ley, 1992 ▸). Diterpenoids isolated from *Salva multicaulis* were reported with significant anti­tuberculosis activity (Ulubelen *et al.*, 1999 ▸). Paclitaxel, a diterpenoid isolated from the *Taxus* genus and which is used in cancer therapy, is recognised as one of the most successful so far in that category (Zhu & Chen, 2019 ▸); forskolin, found in the roots of *Coleus forskohlii*, is a good cardioprotective compound (Jagtap *et al.*, 2011 ▸).

Natural product research has gained more attention due to the failure of other drug discovery techniques to supply lead compounds to face the threatening rise of resistance among cancerous strains, bacteria cells, *etc*. (Butler, 2004 ▸). The extraction of various compounds in different parts of specific plant species has allowed the isolation of compounds with inter­esting biological activities, such as diterpenoids, which have been associated with many therapeutic functions: analgesic, anti-inflammatory, anti­carcinogenic, *etc*. (Sun *et al.*, 2006 ▸).

The objectives of this study were, on one hand, to chromatographically separate and describe the secondary metabolites responsible for the pharmaceutical activities observed during the use of *Staudtia kamerunensis* Warb. (Myristicaceae) in folkloric medicine. Pharmacological studies on the plant highlighted its anti­bacterial properties. Analysis against twelve Gram-negative and Gram-positive microbial strains of the ethyl acetate extract of the stem bark of the specie revealed significant anti­microbial activity, with the lowest MIC being 15.625 µg mL^−1^ (Tonga *et al.*, 2022 ▸). Crystals of Staudtienic acid, isolated from the stem bark, were analysed by single crystal X-ray diffraction to confirm the structure and the stereochemistry of the compound that had been previously published by Noumbissie and collaborators (Noumbissie *et al.*, 1992 ▸). Phytochemists have widely used single-crystal X-ray diffraction to confirm the structures of compounds that were previously characterized using spectroscopic methods (Ahmad *et al.*, 2016 ▸; Simard *et al.*, 2014 ▸; Adelekan *et al.*, 2008 ▸).

We herein report for the first time details of the single-crystal X-ray structure determination of (1*S*,4a*S*,10a*R*)-7-allyl-1,4a-dimethyl-1,2,3,4,4a,9,10,10a-octa­hydro­phenanthrene-1-carb­oxy­lic acid (Staudtienic acid).
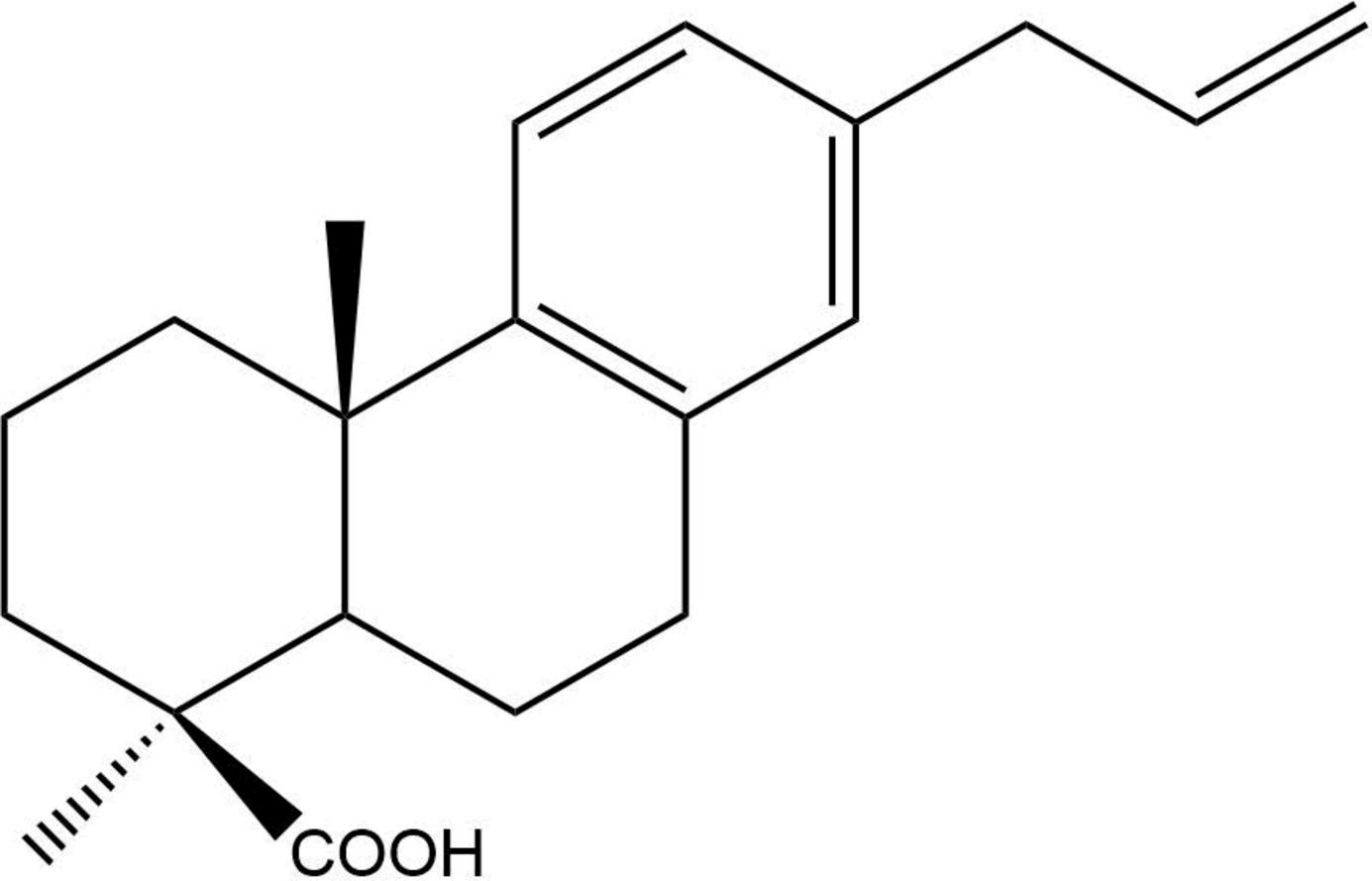


## 2. Structural commentary

**Figure 1:**
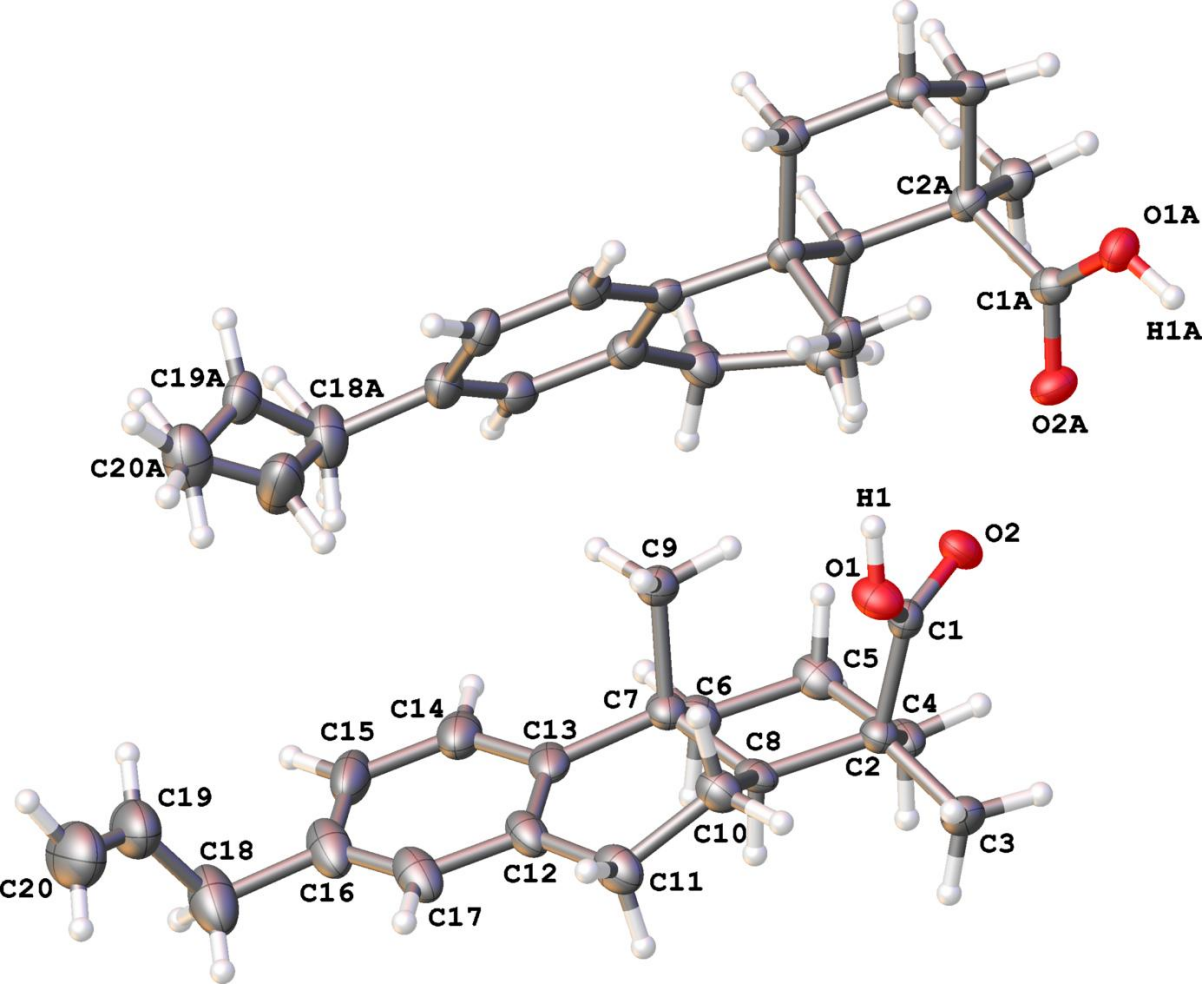
The two independent mol­ecules of the title compound shown with 50% probability ellipsoids.

Plant material was processed as described below, resulting in the title diterpenoid. The title compound crystallizes in the ortho­rhom­bic space group *P*2_1_2_1_2 (No. 18) with two independent mol­ecules in the asymmetric unit (Fig. 1 ▸). Staudtienic acid comprises two fused six-membered rings attached to a benzene. A representation of the mol­ecule with *Mercury* (Mercury 2022.3.0; Macrae *et al.*, 2020 ▸) gives the spatial disposition of the two cyclo­hexane rings. Ring *A* (the ring with the carb­oxy­lic acid) adopts a chair conformation, and ring *B* adopts a half-chair conformation. The half-chair conformation is probably adopted to avoid steric hindrance between the methyl groups and the carb­oxy­lic acid. The crystal structure confirmed the NOE data of the compound previously described by Noumbissie and collaborators (Noumbissie *et al.*, 1992 ▸).

The structure was analysed for unusual geometrical parameters using *Mogul* (Bruno *et al.*, 2004 ▸). Three unusual structural features presented for this material were analysed, relating to the angle around the atom C2. Three angles were classified as unusual as follows: C3—C2—C1 = 104.7 (3)°, C4—C2—C1 = 111.3 (3)° and C8—C2—C1 = 114.6 (3)°. These bond angles deviate from the standard tetra­hedral geometry. The most likely reason for this is probably the presence of the OH groups bonded to C1 that are involved in hydrogen bonding. These may cause small distortions resulting in deviations in the angles around C2.

The propenyl moiety of Mol­ecule *A* is disordered over two positions (see Fig. 1 ▸ for numbering), with the major disorder component residing on C19*A* [66.9 (11) %] and the minor disorder component residing on C19*B* [33.1 (11) %]. Atom H1 was also found to be disordered over two positions, and each atom was assigned a 50% occupancy over O1 and O2. This approach was used as the data were insufficient to determine more precisely the positions of the H atoms from the electron-density map, to place it onto residual electron density, and to refine it to obtain a better percentage occupancy.

## 3. Supra­molecular features

**Figure 2:**
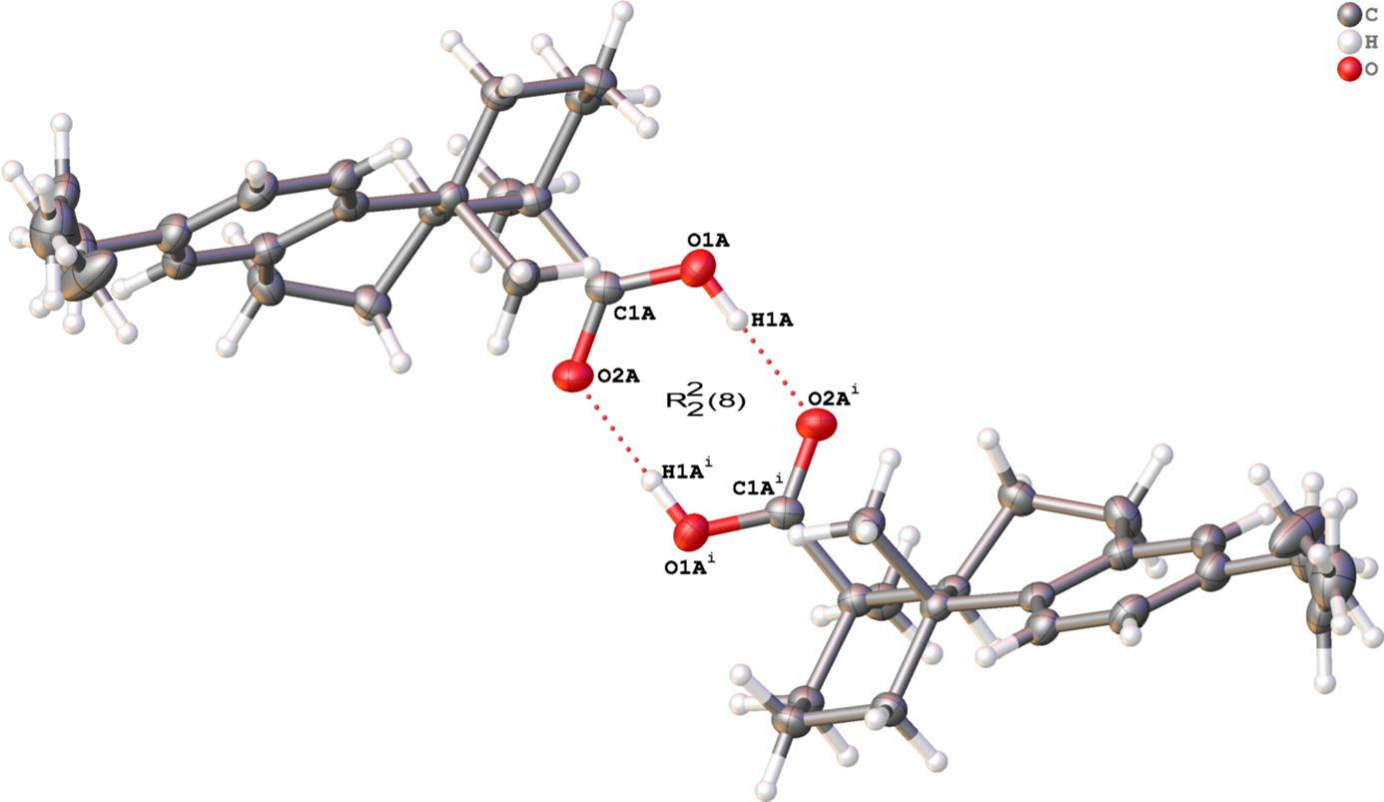
The ring motif of the hydrogen bond between a pair of one of the independent mol­ecules. Hydrogen bonds are denoted as red dotted lines. The graph-set symbol is indicated in the centre of the hydrogen bonding ring; only heteroatoms have been labelled. Symmetry codes: (i) −*x* + 1, −*y* + 1, *z.*

**Figure 3:**
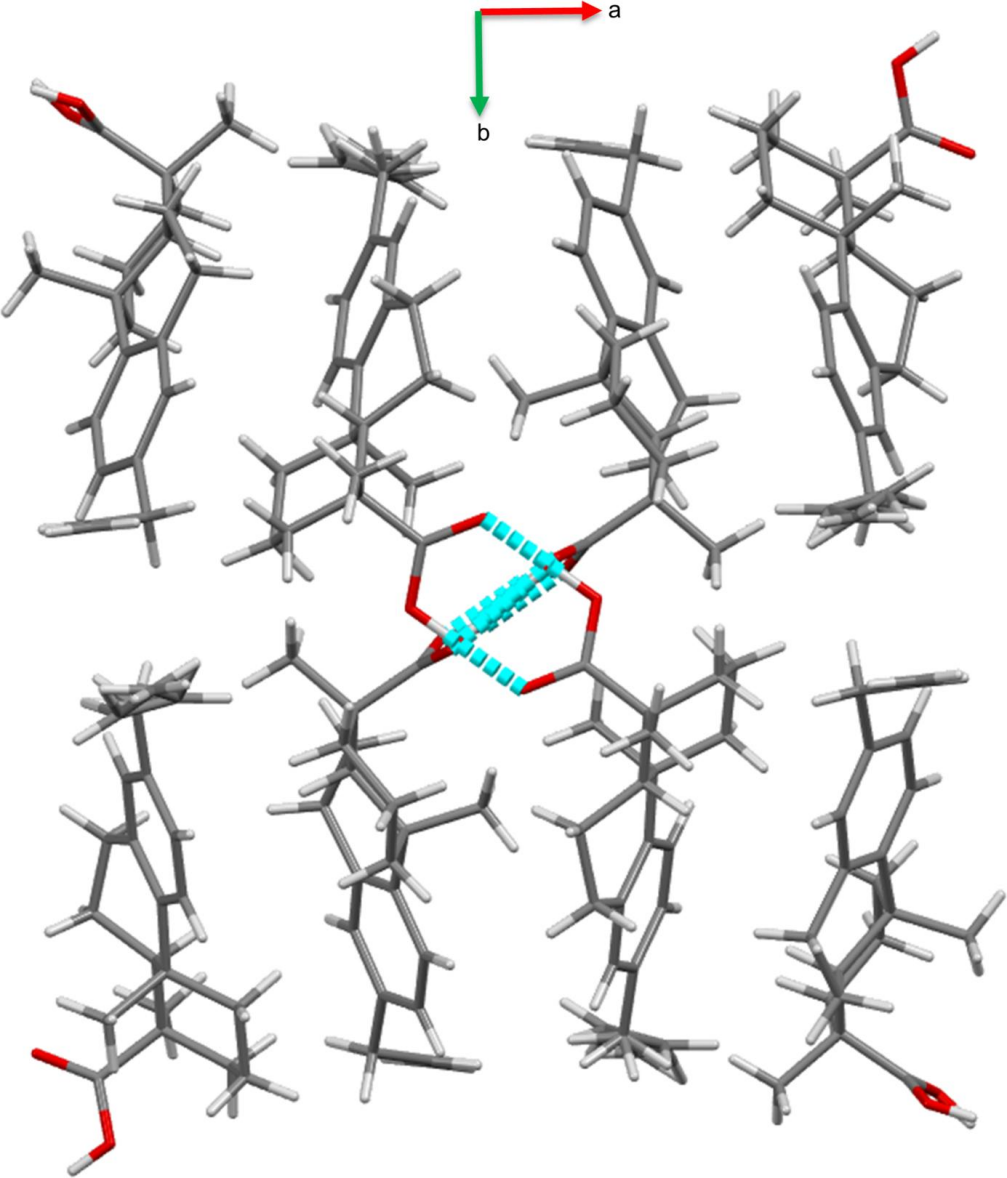
The packing of the title compound viewed down the *c* axis. Hydrogen-bonding contacts are shown in light blue.

Several forms of hydrogen bonding are present in this structure, as shown in Table 1 ▸. The packing of the structure is consolidated by classical O—H⋯O hydrogen bonds as well as by two C—H⋯O inter­molecular hydrogen bonds and two C—H⋯π inter­actions. Each crystallographically independent mol­ecule is connected to another of the same type related by the symmetry operation 1 − *x*, 1 − *y*, *z*. In terms of graph sets (Etter *et al.*, 1990 ▸), this motif is represented by the symbol 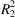
(8) (see Fig. 2 ▸). The two dimers are then connected by C—H⋯O hydrogen bonds, forming an infinite column along the *c*-axis. The columns are connected to similar columns by van der Waals inter­actions with mol­ecules related by 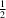 + *x*, 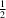 − *y*, 1 − *z* and 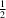 − *x*, −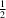 + *y*, 1 − *z*. The most illustrative view of the packing arrangement of the title compound is the view down the *c* axis shown in Fig. 3 ▸.

## 4. Database survey

The structure was thoroughly validated using *PLATON* (Spek, 2020 ▸). A search was also performed in the Cambridge Structural Database (CSD Version 5.44 2023.1, March 2023 update; Groom *et al.*, 2016 ▸), and it was established that this structure had not been previously published or deposited.

A search in the CSD for organic compounds with the backbone of two fused rings attached to a benzene revealed 1390 hits. When the propenyl substituent of the benzene ring is introduced, the number of hits is reduced to four [refcodes LATPOP (Zhu *et al.*, 2022 ▸), LAPHOA (Hitchcock *et al.*, 2005 ▸), MIXFUX (Ye *et al.*, 2019 ▸) and OVAQIN (Green *et al.*, 2016 ▸)]. When the carb­oxy­lic functional group search is introduced at position C4 of the cyclo­hexane, the number of search hits is reduced to zero.

## 5. Synthesis and crystallization

**Plant Material.** The stem bark of this plant species was collected from Minkam Mengale Menkom, a location in the South region of Cameroon. A voucher specimen is in deposit there under the number 49184 HNC.

**Extraction and Isolation.** Extraction and isolation were carried out as described by Noumbissie and collaborators (Noumbissie *et al.*, 1992 ▸). Briefly, after collection, the sample was dried and ground to yield a powder of 15.79 Kg. A Soxhlet apparatus was used to extract the stem bark using ethyl acetate, and the liquor obtained was evaporated using a rotary evaporator under reduced pressure. A residue of mass 550.12 grams was obtained, and 250 g was partitioned using benzene (C_6_H_6_) to obtain 41.96 g (yield 41.86, 16.74%) of the sample. The latter was loaded with 500 g of silica, and elution was done with a gradient of polarity starting with benzene, then a mixture of benzene and petroleum ether, then petroleum ether. The final sample was obtained from a mixture of 60% petroleum ether in benzene (yield: 80.22 mg, 0.19%)

## 6. Refinement

The systematic absences in the diffraction data were uniquely consistent with the space group *P*2_1_2_1_2 (No. 18) determined by *XPREP* (Bruker, 2014 ▸), which yielded chemically reasonable and computationally stable results of refinement (Sheldrick, 2015*a* ▸,*b* ▸).

A successful solution by Intrinsic phasing methods (*SHELXT;* Sheldrick, 2015*a* ▸) provided all non-hydrogen atoms from the *E*-map. The remaining hydrogen atoms were located in an alternating series of least-squares cycles and visualization of the difference-Fourier map. All non-hydrogen atoms were refined with anisotropic displacement coefficients. All hydrogen atoms connected to C atoms were included in the structure-factor calculation at idealized positions and were allowed to ride on the neighbouring atoms with relative isotropic displacement coefficients. Two symmetry-independent mol­ecules are in the asymmetric unit as shown in Fig. 1 ▸.

The compound crystallized in a chiral space group and the Flack *x* parameter was determined using 1758 quotients (Parsons *et al.*, 2013 ▸). However, since the data were collected using Mo radiation, the absolute structure could not be reliably determined. In addition, the crystals used for the data collection diffracted weakly. Attempts were made to find crystal structures of similar compounds where the structural chirality was already known, but these attempts were unsuccessful, as no hits could be found in the CSD.

The final least-squares refinement of 420 parameters against 8260 reflections resulted in residuals *R* (based on *F*^2^ for *I*≥2*σ*) and *wR* (based on *F*^2^ for all data) of 0.0522 and 0.1339, respectively. The final difference-Fourier map was basically featureless. The structure was validated using *PLATON* (Spek, 2020 ▸). Crystal data, data collection and structure refinement details are summarized in Table 2 ▸.

## Supplementary Material

Crystal structure: contains datablock(s) I. DOI: 10.1107/S2056989024005000/dj2072sup1.cif

Structure factors: contains datablock(s) I. DOI: 10.1107/S2056989024005000/dj2072Isup2.hkl

Supporting information file. DOI: 10.1107/S2056989024005000/dj2072Isup3.cml

CCDC reference: 2358697

Additional supporting information:  crystallographic information; 3D view; checkCIF report

## Figures and Tables

**Table 1:** Hydrogen-bond geometry (Å, °) *Cg*3 and *Cg*6 are the centroids of the C12*A*–C17*A* and C12–C17 rings, respectively.

*D*—H⋯*A*	*D*—H	H⋯*A*	*D*⋯*A*	*D*—H⋯*A*
O1—H1⋯O1^i^	0.87 (2)	1.88 (3)	2.720 (5)	161 (8)
O2—H2⋯O2^i^	0.87 (2)	1.82 (3)	2.656 (5)	162 (7)
O1*A*—H1*A*⋯O2*A*^i^	0.87 (2)	1.78 (2)	2.652 (3)	174 (4)
C10*A*—H10*B*⋯O2*A*	0.99	2.51	2.917 (4)	104
C10—H10*D*⋯O1	0.99	2.58	2.969 (4)	104
C4—H4*B*⋯*Cg*3^ii^	0.99	3.00	3.978 (4)	170
C4*A*—H4*AB*⋯*Cg*6^iii^	0.99	2.90	3.863 (4)	165

Symmetry codes: (i) 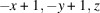; (ii) 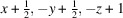; (iii) 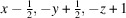.

**Table 2:** Experimental details

Crystal data	
Chemical formula	C_20_H_26_O_2_
*M*_r_	298.41
Crystal system, space group	Orthorhombic, *P*2_1_2_1_2
Temperature (K)	100
*a*, *b*, *c* (Å)	18.396 (3), 20.248 (4), 8.9338 (16)
*V* (Å^3^)	3327.8 (10)
*Z*	8
Radiation type	Mo *K*α
μ (mm^−1^)	0.08
Crystal size (mm)	0.75 × 0.16 × 0.06

Data collection	
Diffractometer	Bruker APEXII CCD
Absorption correction	Multi-scan (*SADABS*; Krause *et al.*, 2015 ▸)
*T*_min_, *T*_max_	0.670, 0.746
No. of measured, independent and observed [*I* > 2σ(*I*)] reflections	30395, 8260, 5374
*R*_int_	0.087
(sin θ/λ)_max_ (Å^−1^)	0.667

Refinement	
*R*[*F*^2^ > 2σ(*F*^2^)], *wR*(*F*^2^), *S*	0.055, 0.134, 1.02
No. of reflections	8260
No. of parameters	420
No. of restraints	20
H-atom treatment	H atoms treated by a mixture of independent and constrained refinement
Δρ_max_, Δρ_min_ (e Å^−3^)	0.35, −0.25
Absolute structure	Flack *x* determined using 1758 quotients [(*I*^+^)−(*I*^−^)]/[(*I*^+^)+(*I*^−^)] (Parsons *et al.*, 2013 ▸)
Absolute structure parameter	−1.0 (9)

Computer programs: *APEX2* and *SAINT* (Bruker, 2014 ▸), *SHELXT* (Sheldrick, 2015*a* ▸), *SHELXL2019/2* (Sheldrick, 2015*b* ▸) and *OLEX2* (Dolomanov *et al.*, 2009 ▸).

## supplementary crystallographic information

### (1S,4aS,10aR)-1,4a-Dimethyl-7-(prop-2-en-1-yl)-1,2,3,4,4a,9,10,10a-octahydrophenanthrene-1-carboxylic acid Crystal data

**Table:**

C_20_H_26_O_2_	*D*_x_ = 1.191 Mg m^−^^3^
*M**_r_* = 298.41	Mo *K*α radiation, λ = 0.71073 Å
Orthorhombic, *P*2_1_2_1_2	Cell parameters from 2900 reflections
*a* = 18.396 (3) Å	θ = 2.3–21.4°
*b* = 20.248 (4) Å	µ = 0.08 mm^−^^1^
*c* = 8.9338 (16) Å	*T* = 100 K
*V* = 3327.8 (10) Å^3^	Rod, colourless
*Z* = 8	0.75 × 0.16 × 0.06 mm
*F*(000) = 1296	

### (1S,4aS,10aR)-1,4a-Dimethyl-7-(prop-2-en-1-yl)-1,2,3,4,4a,9,10,10a-octahydrophenanthrene-1-carboxylic acid Data collection

**Table:**

Bruker APEXII CCD diffractometer	5374 reflections with *I* > 2σ(*I*)
φ and ω scans	*R*_int_ = 0.087
Absorption correction: multi-scan (SADABS; Krause *et al.*, 2015)	θ_max_ = 28.3°, θ_min_ = 2.3°
*T*_min_ = 0.670, *T*_max_ = 0.746	*h* = −24→24
30395 measured reflections	*k* = −26→27
8260 independent reflections	*l* = −11→11

### (1S,4aS,10aR)-1,4a-Dimethyl-7-(prop-2-en-1-yl)-1,2,3,4,4a,9,10,10a-octahydrophenanthrene-1-carboxylic acid Refinement

**Table:**

Refinement on *F*^2^	Hydrogen site location: mixed
Least-squares matrix: full	H atoms treated by a mixture of independent and constrained refinement
*R*[*F*^2^ > 2σ(*F*^2^)] = 0.055	*w* = 1/[σ^2^(*F*_o_^2^) + (0.057*P*)^2^ + 0.2719*P*] where *P* = (*F*_o_^2^ + 2*F*_c_^2^)/3
*wR*(*F*^2^) = 0.134	(Δ/σ)_max_ < 0.001
*S* = 1.02	Δρ_max_ = 0.35 e Å^−^^3^
8260 reflections	Δρ_min_ = −0.25 e Å^−^^3^
420 parameters	Absolute structure: Flack *x* determined using 1758 quotients [(*I*^+^)-(*I*^-^)]/[(*I*^+^)+(*I*^-^)] (Parsons *et al.*, 2013)
20 restraints	Absolute structure parameter: −1.0 (9)
Primary atom site location: structure-invariant direct methods	

### (1S,4aS,10aR)-1,4a-Dimethyl-7-(prop-2-en-1-yl)-1,2,3,4,4a,9,10,10a-octahydrophenanthrene-1-carboxylic acid Special details

**Table:**

Geometry. All esds (except the esd in the dihedral angle between two l.s. planes) are estimated using the full covariance matrix. The cell esds are taken into account individually in the estimation of esds in distances, angles and torsion angles; correlations between esds in cell parameters are only used when they are defined by crystal symmetry. An approximate (isotropic) treatment of cell esds is used for estimating esds involving l.s. planes.

### (1S,4aS,10aR)-1,4a-Dimethyl-7-(prop-2-en-1-yl)-1,2,3,4,4a,9,10,10a-octahydrophenanthrene-1-carboxylic acid Fractional atomic coordinates and isotropic or equivalent isotropic displacement parameters (Å^2^)

**Table:**

	*x*	*y*	*z*	*U*_iso_*/*U*_eq_	Occ. (<1)
O1	0.56424 (13)	0.46675 (12)	0.7674 (3)	0.0332 (6)	
H1	0.5190 (18)	0.480 (4)	0.774 (9)	0.050*	0.5
O2	0.55451 (13)	0.45701 (11)	0.5195 (3)	0.0284 (6)	
H2	0.521 (4)	0.485 (3)	0.540 (8)	0.043*	0.5
C1	0.58554 (18)	0.44326 (15)	0.6431 (4)	0.0242 (7)	
C2	0.65409 (18)	0.40062 (15)	0.6393 (4)	0.0248 (7)	
C3	0.71745 (19)	0.44981 (18)	0.6655 (4)	0.0340 (9)	
H3A	0.718685	0.482103	0.583872	0.051*	
H3B	0.763564	0.425609	0.668450	0.051*	
H3C	0.710142	0.472805	0.760888	0.051*	
C4	0.66498 (19)	0.36895 (17)	0.4844 (4)	0.0297 (8)	
H4A	0.658364	0.403283	0.406667	0.036*	
H4B	0.715551	0.352570	0.476945	0.036*	
C5	0.6134 (2)	0.31232 (17)	0.4524 (4)	0.0318 (8)	
H5A	0.626279	0.292022	0.355183	0.038*	
H5B	0.563210	0.329632	0.444528	0.038*	
C6	0.6161 (2)	0.25967 (17)	0.5745 (4)	0.0317 (8)	
H6A	0.664588	0.238388	0.573209	0.038*	
H6B	0.579441	0.225281	0.552315	0.038*	
C7	0.60174 (18)	0.28782 (16)	0.7327 (4)	0.0266 (8)	
C8	0.65639 (18)	0.34497 (15)	0.7594 (4)	0.0248 (7)	
H8	0.705269	0.324129	0.746452	0.030*	
C9	0.52197 (18)	0.31055 (17)	0.7437 (4)	0.0301 (8)	
H9A	0.513496	0.330801	0.841793	0.045*	
H9B	0.489747	0.272387	0.731479	0.045*	
H9C	0.511930	0.342923	0.664809	0.045*	
C10	0.6548 (2)	0.36695 (17)	0.9240 (4)	0.0309 (8)	
H10C	0.684534	0.407317	0.936953	0.037*	
H10D	0.604316	0.377093	0.954461	0.037*	
C11	0.6850 (2)	0.31163 (19)	1.0201 (4)	0.0371 (9)	
H11C	0.737941	0.307626	1.001996	0.045*	
H11D	0.677905	0.322860	1.126967	0.045*	
C12	0.6492 (2)	0.24637 (18)	0.9880 (4)	0.0341 (9)	
C13	0.61215 (19)	0.23419 (17)	0.8535 (5)	0.0324 (8)	
C14	0.5810 (2)	0.17213 (18)	0.8346 (5)	0.0416 (10)	
H14	0.555181	0.162760	0.745003	0.050*	
C15	0.5869 (2)	0.12301 (19)	0.9450 (6)	0.0479 (11)	
H15	0.565106	0.081078	0.928701	0.057*	
C16	0.6239 (2)	0.1348 (2)	1.0767 (5)	0.0474 (11)	
C17	0.6539 (2)	0.19641 (19)	1.0967 (4)	0.0429 (10)	
H17	0.678779	0.205523	1.187577	0.051*	
C18	0.6342 (3)	0.0809 (2)	1.1954 (5)	0.0614 (14)	
H18E	0.642602	0.038240	1.143999	0.074*	
H18F	0.678417	0.091157	1.254243	0.074*	
C19	0.5741 (3)	0.0733 (2)	1.2969 (6)	0.0616 (14)	
H19	0.527301	0.069147	1.252872	0.074*	
C20	0.5768 (3)	0.0716 (3)	1.4410 (6)	0.0705 (15)	
H20E	0.622148	0.075599	1.490870	0.085*	
H20F	0.533331	0.066420	1.497293	0.085*	
O1A	0.40394 (13)	0.50455 (12)	0.1249 (3)	0.0295 (6)	
H1A	0.4424 (15)	0.5296 (17)	0.119 (4)	0.044*	
O2A	0.47966 (12)	0.42012 (12)	0.0856 (3)	0.0310 (6)	
C1A	0.41645 (18)	0.44294 (16)	0.1014 (3)	0.0239 (7)	
C2A	0.35119 (18)	0.39732 (16)	0.0836 (4)	0.0240 (7)	
C3A	0.34903 (19)	0.38042 (17)	−0.0849 (4)	0.0289 (8)	
H3AA	0.339858	0.420707	−0.142653	0.043*	
H3AB	0.310110	0.348443	−0.103780	0.043*	
H3AC	0.395753	0.361376	−0.115109	0.043*	
C4A	0.27859 (17)	0.43218 (17)	0.1205 (4)	0.0248 (7)	
H4AA	0.278072	0.475876	0.070739	0.030*	
H4AB	0.238208	0.405831	0.078033	0.030*	
C5A	0.26492 (18)	0.44198 (17)	0.2872 (4)	0.0268 (8)	
H5AA	0.215456	0.460090	0.302038	0.032*	
H5AB	0.300194	0.474431	0.327153	0.032*	
C6A	0.27216 (17)	0.37731 (16)	0.3734 (4)	0.0258 (7)	
H6AA	0.233167	0.346746	0.341191	0.031*	
H6AB	0.265497	0.386121	0.481498	0.031*	
C7A	0.34653 (17)	0.34383 (15)	0.3490 (4)	0.0218 (7)	
C8A	0.35657 (17)	0.33317 (16)	0.1781 (3)	0.0223 (7)	
H8A	0.313473	0.306353	0.147075	0.027*	
C9A	0.40633 (18)	0.38853 (17)	0.4169 (4)	0.0275 (8)	
H9AA	0.454279	0.371722	0.388055	0.041*	
H9AB	0.402091	0.388446	0.526269	0.041*	
H9AC	0.400462	0.433702	0.379449	0.041*	
C10A	0.42229 (18)	0.28852 (16)	0.1463 (4)	0.0278 (7)	
H10A	0.431446	0.286469	0.037178	0.033*	
H10B	0.466155	0.306707	0.195500	0.033*	
C11A	0.4064 (2)	0.21939 (17)	0.2065 (4)	0.0331 (9)	
H11A	0.368715	0.198306	0.143358	0.040*	
H11B	0.451019	0.192244	0.199876	0.040*	
C12A	0.38058 (18)	0.22087 (17)	0.3665 (4)	0.0270 (8)	
C13A	0.35080 (18)	0.27748 (15)	0.4319 (4)	0.0239 (7)	
C14A	0.32951 (18)	0.27379 (16)	0.5820 (4)	0.0276 (8)	
H14A	0.309260	0.311797	0.628374	0.033*	
C15A	0.33707 (19)	0.21632 (17)	0.6654 (4)	0.0310 (8)	
H15A	0.322021	0.215635	0.767102	0.037*	
C16A	0.36640 (19)	0.15989 (17)	0.6013 (4)	0.0312 (8)	
C17A	0.38702 (19)	0.16302 (17)	0.4524 (4)	0.0314 (8)	
H17A	0.406303	0.124501	0.406476	0.038*	
C18A	0.3772 (3)	0.0960 (2)	0.6893 (5)	0.0479 (11)	
H18A	0.365848	0.058535	0.622049	0.057*	0.669 (11)
H18B	0.429247	0.092575	0.716117	0.057*	0.669 (11)
H18C	0.332193	0.069420	0.680638	0.057*	0.331 (11)
H18D	0.416742	0.070588	0.641157	0.057*	0.331 (11)
C19A	0.3349 (3)	0.0880 (2)	0.8249 (6)	0.0337 (17)	0.669 (11)
H19A	0.283597	0.088146	0.813523	0.040*	0.669 (11)
C19B	0.3942 (7)	0.1029 (7)	0.8450 (8)	0.061 (5)	0.331 (11)
H19B	0.437014	0.127386	0.866117	0.073*	0.331 (11)
C20A	0.3597 (3)	0.0805 (2)	0.9620 (5)	0.0525 (12)	
H20A	0.410601	0.080027	0.979977	0.063*	0.669 (11)
H20B	0.326695	0.075693	1.043019	0.063*	0.669 (11)
H20C	0.316391	0.055587	0.949704	0.063*	0.331 (11)
H20D	0.377897	0.089238	1.059547	0.063*	0.331 (11)

### (1S,4aS,10aR)-1,4a-Dimethyl-7-(prop-2-en-1-yl)-1,2,3,4,4a,9,10,10a-octahydrophenanthrene-1-carboxylic acid Atomic displacement parameters (Å^2^)

**Table:**

	*U*^11^	*U*^22^	*U*^33^	*U*^12^	*U*^13^	*U*^23^
O1	0.0327 (14)	0.0355 (14)	0.0313 (14)	0.0117 (11)	0.0014 (12)	−0.0021 (12)
O2	0.0278 (14)	0.0245 (12)	0.0328 (14)	0.0044 (10)	−0.0060 (11)	0.0023 (11)
C1	0.0232 (17)	0.0220 (16)	0.0274 (18)	−0.0009 (13)	0.0030 (16)	0.0034 (15)
C2	0.0224 (16)	0.0251 (16)	0.0270 (17)	0.0038 (14)	0.0012 (16)	0.0028 (15)
C3	0.0244 (18)	0.036 (2)	0.042 (2)	−0.0023 (15)	−0.0028 (17)	0.0071 (18)
C4	0.0251 (19)	0.0339 (19)	0.0299 (19)	0.0088 (15)	0.0068 (16)	0.0064 (16)
C5	0.035 (2)	0.0336 (19)	0.0266 (19)	0.0103 (16)	0.0044 (17)	−0.0039 (16)
C6	0.033 (2)	0.0258 (17)	0.037 (2)	0.0051 (14)	0.0025 (17)	−0.0009 (16)
C7	0.0235 (18)	0.0260 (17)	0.0303 (19)	0.0057 (14)	0.0051 (15)	0.0031 (16)
C8	0.0211 (17)	0.0275 (17)	0.0259 (17)	0.0090 (14)	0.0009 (15)	0.0008 (15)
C9	0.0252 (18)	0.0271 (17)	0.038 (2)	0.0021 (14)	0.0028 (17)	0.0026 (16)
C10	0.032 (2)	0.0334 (19)	0.0271 (18)	0.0059 (16)	0.0001 (17)	0.0010 (16)
C11	0.041 (2)	0.044 (2)	0.026 (2)	0.0107 (17)	0.0043 (17)	0.0041 (18)
C12	0.037 (2)	0.0344 (19)	0.031 (2)	0.0142 (17)	0.0090 (19)	0.0086 (16)
C13	0.0243 (18)	0.0304 (18)	0.043 (2)	0.0086 (15)	0.0133 (18)	0.0082 (17)
C14	0.033 (2)	0.034 (2)	0.058 (3)	0.0050 (16)	0.012 (2)	0.009 (2)
C15	0.041 (2)	0.0275 (19)	0.075 (3)	0.0024 (17)	0.020 (2)	0.013 (2)
C16	0.056 (3)	0.043 (2)	0.044 (2)	0.019 (2)	0.023 (2)	0.014 (2)
C17	0.052 (3)	0.042 (2)	0.034 (2)	0.020 (2)	0.014 (2)	0.0077 (19)
C18	0.080 (4)	0.047 (3)	0.057 (3)	0.019 (2)	0.024 (3)	0.017 (2)
C19	0.054 (3)	0.068 (3)	0.062 (3)	0.005 (2)	0.009 (3)	0.028 (3)
C20	0.069 (4)	0.088 (4)	0.055 (3)	0.003 (3)	0.012 (3)	0.021 (3)
O1A	0.0276 (13)	0.0253 (12)	0.0356 (14)	−0.0042 (10)	0.0028 (12)	0.0012 (11)
O2A	0.0210 (12)	0.0325 (13)	0.0393 (14)	−0.0003 (10)	0.0038 (11)	−0.0017 (12)
C1A	0.0219 (17)	0.0304 (18)	0.0195 (17)	0.0009 (13)	0.0002 (14)	0.0004 (14)
C2A	0.0223 (17)	0.0266 (16)	0.0232 (17)	−0.0010 (14)	0.0006 (15)	−0.0009 (14)
C3A	0.0275 (18)	0.0347 (18)	0.0246 (17)	−0.0007 (15)	0.0009 (16)	−0.0009 (16)
C4A	0.0200 (16)	0.0257 (16)	0.0287 (19)	0.0009 (13)	−0.0021 (15)	0.0001 (15)
C5A	0.0207 (17)	0.0304 (19)	0.0291 (18)	0.0040 (13)	0.0034 (15)	−0.0014 (16)
C6A	0.0234 (17)	0.0266 (17)	0.0276 (18)	0.0010 (13)	0.0030 (15)	−0.0030 (16)
C7A	0.0205 (16)	0.0224 (15)	0.0224 (16)	−0.0020 (13)	−0.0015 (15)	−0.0015 (14)
C8A	0.0191 (16)	0.0245 (16)	0.0232 (17)	−0.0024 (13)	−0.0003 (14)	−0.0018 (14)
C9A	0.0273 (18)	0.0280 (17)	0.0273 (18)	−0.0046 (14)	−0.0036 (15)	0.0008 (15)
C10A	0.0260 (18)	0.0303 (18)	0.0272 (17)	0.0050 (14)	0.0028 (16)	−0.0027 (16)
C11A	0.037 (2)	0.0276 (18)	0.035 (2)	0.0073 (15)	0.0019 (17)	0.0008 (16)
C12A	0.0220 (17)	0.0272 (17)	0.0319 (19)	−0.0016 (13)	0.0004 (16)	0.0004 (16)
C13A	0.0183 (16)	0.0262 (16)	0.0271 (17)	−0.0031 (13)	−0.0042 (15)	−0.0011 (14)
C14A	0.0266 (19)	0.0256 (17)	0.0307 (19)	−0.0042 (14)	0.0007 (15)	−0.0047 (15)
C15A	0.031 (2)	0.0330 (19)	0.0287 (19)	−0.0090 (15)	0.0031 (16)	0.0031 (16)
C16A	0.031 (2)	0.0272 (18)	0.035 (2)	−0.0047 (14)	0.0006 (16)	0.0051 (16)
C17A	0.0265 (18)	0.0242 (17)	0.044 (2)	−0.0008 (14)	0.0003 (17)	0.0014 (17)
C18A	0.063 (3)	0.032 (2)	0.048 (2)	0.0002 (19)	0.010 (2)	0.0080 (19)
C19A	0.039 (4)	0.022 (3)	0.040 (3)	−0.005 (2)	0.007 (3)	0.000 (2)
C19B	0.055 (9)	0.066 (9)	0.063 (6)	−0.024 (7)	−0.015 (6)	0.026 (6)
C20A	0.073 (3)	0.041 (2)	0.044 (2)	−0.007 (2)	−0.005 (2)	0.003 (2)

### (1S,4aS,10aR)-1,4a-Dimethyl-7-(prop-2-en-1-yl)-1,2,3,4,4a,9,10,10a-octahydrophenanthrene-1-carboxylic acid Geometric parameters (Å, º)

**Table:**

O1—H1	0.87 (2)	C1A—C2A	1.523 (5)
O1—C1	1.270 (4)	C2A—C3A	1.544 (5)
O2—H2	0.87 (2)	C2A—C4A	1.546 (4)
O2—C1	1.274 (4)	C2A—C8A	1.552 (4)
C1—C2	1.529 (4)	C3A—H3AA	0.9800
C2—C3	1.551 (5)	C3A—H3AB	0.9800
C2—C4	1.538 (5)	C3A—H3AC	0.9800
C2—C8	1.557 (5)	C4A—H4AA	0.9900
C3—H3A	0.9800	C4A—H4AB	0.9900
C3—H3B	0.9800	C4A—C5A	1.524 (5)
C3—H3C	0.9800	C5A—H5AA	0.9900
C4—H4A	0.9900	C5A—H5AB	0.9900
C4—H4B	0.9900	C5A—C6A	1.525 (5)
C4—C5	1.516 (5)	C6A—H6AA	0.9900
C5—H5A	0.9900	C6A—H6AB	0.9900
C5—H5B	0.9900	C6A—C7A	1.542 (4)
C5—C6	1.526 (5)	C7A—C8A	1.553 (4)
C6—H6A	0.9900	C7A—C9A	1.548 (4)
C6—H6B	0.9900	C7A—C13A	1.536 (4)
C6—C7	1.547 (5)	C8A—H8A	1.0000
C7—C8	1.551 (5)	C8A—C10A	1.536 (4)
C7—C9	1.541 (5)	C9A—H9AA	0.9800
C7—C13	1.543 (5)	C9A—H9AB	0.9800
C8—H8	1.0000	C9A—H9AC	0.9800
C8—C10	1.536 (5)	C10A—H10A	0.9900
C9—H9A	0.9800	C10A—H10B	0.9900
C9—H9B	0.9800	C10A—C11A	1.528 (5)
C9—H9C	0.9800	C11A—H11A	0.9900
C10—H10C	0.9900	C11A—H11B	0.9900
C10—H10D	0.9900	C11A—C12A	1.507 (5)
C10—C11	1.517 (5)	C12A—C13A	1.398 (5)
C11—H11C	0.9900	C12A—C17A	1.405 (5)
C11—H11D	0.9900	C13A—C14A	1.400 (5)
C11—C12	1.504 (6)	C14A—H14A	0.9500
C12—C13	1.403 (6)	C14A—C15A	1.389 (5)
C12—C17	1.405 (5)	C15A—H15A	0.9500
C13—C14	1.391 (5)	C15A—C16A	1.387 (5)
C14—H14	0.9500	C16A—C17A	1.385 (5)
C14—C15	1.405 (6)	C16A—C18A	1.527 (5)
C15—H15	0.9500	C17A—H17A	0.9500
C15—C16	1.380 (6)	C18A—H18A	0.9900
C16—C17	1.376 (6)	C18A—H18B	0.9900
C16—C18	1.534 (6)	C18A—H18C	0.9900
C17—H17	0.9500	C18A—H18D	0.9900
C18—H18E	0.9900	C18A—C19A	1.448 (6)
C18—H18F	0.9900	C18A—C19B	1.432 (8)
C18—C19	1.439 (7)	C19A—H19A	0.9500
C19—H19	0.9500	C19A—C20A	1.316 (6)
C19—C20	1.289 (7)	C19B—H19B	0.9500
C20—H20E	0.9500	C19B—C20A	1.304 (8)
C20—H20F	0.9500	C20A—H20A	0.9500
O1A—H1A	0.872 (19)	C20A—H20B	0.9500
O1A—C1A	1.286 (4)	C20A—H20C	0.9500
O2A—C1A	1.259 (4)	C20A—H20D	0.9500
			
C1—O1—H1	118 (5)	C1A—C2A—C8A	113.6 (3)
C1—O2—H2	106 (5)	C3A—C2A—C4A	106.7 (3)
O1—C1—O2	122.5 (3)	C3A—C2A—C8A	110.3 (3)
O1—C1—C2	119.0 (3)	C4A—C2A—C8A	108.7 (3)
O2—C1—C2	118.3 (3)	C2A—C3A—H3AA	109.5
C1—C2—C3	104.7 (3)	C2A—C3A—H3AB	109.5
C1—C2—C4	111.3 (3)	C2A—C3A—H3AC	109.5
C1—C2—C8	114.6 (3)	H3AA—C3A—H3AB	109.5
C3—C2—C8	109.9 (3)	H3AA—C3A—H3AC	109.5
C4—C2—C3	107.8 (3)	H3AB—C3A—H3AC	109.5
C4—C2—C8	108.4 (3)	C2A—C4A—H4AA	108.7
C2—C3—H3A	109.5	C2A—C4A—H4AB	108.7
C2—C3—H3B	109.5	H4AA—C4A—H4AB	107.6
C2—C3—H3C	109.5	C5A—C4A—C2A	114.2 (3)
H3A—C3—H3B	109.5	C5A—C4A—H4AA	108.7
H3A—C3—H3C	109.5	C5A—C4A—H4AB	108.7
H3B—C3—H3C	109.5	C4A—C5A—H5AA	109.3
C2—C4—H4A	108.8	C4A—C5A—H5AB	109.3
C2—C4—H4B	108.8	C4A—C5A—C6A	111.5 (3)
H4A—C4—H4B	107.7	H5AA—C5A—H5AB	108.0
C5—C4—C2	113.8 (3)	C6A—C5A—H5AA	109.3
C5—C4—H4A	108.8	C6A—C5A—H5AB	109.3
C5—C4—H4B	108.8	C5A—C6A—H6AA	109.1
C4—C5—H5A	109.2	C5A—C6A—H6AB	109.1
C4—C5—H5B	109.2	C5A—C6A—C7A	112.6 (3)
C4—C5—C6	111.9 (3)	H6AA—C6A—H6AB	107.8
H5A—C5—H5B	107.9	C7A—C6A—H6AA	109.1
C6—C5—H5A	109.2	C7A—C6A—H6AB	109.1
C6—C5—H5B	109.2	C6A—C7A—C8A	107.8 (3)
C5—C6—H6A	109.0	C6A—C7A—C9A	108.5 (3)
C5—C6—H6B	109.0	C9A—C7A—C8A	112.5 (3)
C5—C6—C7	112.9 (3)	C13A—C7A—C6A	111.2 (3)
H6A—C6—H6B	107.8	C13A—C7A—C8A	110.3 (3)
C7—C6—H6A	109.0	C13A—C7A—C9A	106.6 (3)
C7—C6—H6B	109.0	C2A—C8A—C7A	114.2 (3)
C6—C7—C8	107.7 (3)	C2A—C8A—H8A	104.7
C9—C7—C6	109.4 (3)	C7A—C8A—H8A	104.7
C9—C7—C8	112.6 (3)	C10A—C8A—C2A	116.2 (3)
C9—C7—C13	106.5 (3)	C10A—C8A—C7A	110.9 (3)
C13—C7—C6	111.0 (3)	C10A—C8A—H8A	104.7
C13—C7—C8	109.7 (3)	C7A—C9A—H9AA	109.5
C2—C8—H8	104.5	C7A—C9A—H9AB	109.5
C7—C8—C2	114.6 (3)	C7A—C9A—H9AC	109.5
C7—C8—H8	104.5	H9AA—C9A—H9AB	109.5
C10—C8—C2	116.7 (3)	H9AA—C9A—H9AC	109.5
C10—C8—C7	110.6 (3)	H9AB—C9A—H9AC	109.5
C10—C8—H8	104.5	C8A—C10A—H10A	109.9
C7—C9—H9A	109.5	C8A—C10A—H10B	109.9
C7—C9—H9B	109.5	H10A—C10A—H10B	108.3
C7—C9—H9C	109.5	C11A—C10A—C8A	108.9 (3)
H9A—C9—H9B	109.5	C11A—C10A—H10A	109.9
H9A—C9—H9C	109.5	C11A—C10A—H10B	109.9
H9B—C9—H9C	109.5	C10A—C11A—H11A	109.2
C8—C10—H10C	109.9	C10A—C11A—H11B	109.2
C8—C10—H10D	109.9	H11A—C11A—H11B	107.9
H10C—C10—H10D	108.3	C12A—C11A—C10A	112.1 (3)
C11—C10—C8	108.7 (3)	C12A—C11A—H11A	109.2
C11—C10—H10C	109.9	C12A—C11A—H11B	109.2
C11—C10—H10D	109.9	C13A—C12A—C11A	122.4 (3)
C10—C11—H11C	109.1	C13A—C12A—C17A	119.2 (3)
C10—C11—H11D	109.1	C17A—C12A—C11A	118.4 (3)
H11C—C11—H11D	107.9	C12A—C13A—C7A	122.4 (3)
C12—C11—C10	112.3 (3)	C12A—C13A—C14A	117.8 (3)
C12—C11—H11C	109.1	C14A—C13A—C7A	119.6 (3)
C12—C11—H11D	109.1	C13A—C14A—H14A	119.0
C13—C12—C11	122.0 (3)	C15A—C14A—C13A	122.1 (3)
C13—C12—C17	119.7 (4)	C15A—C14A—H14A	119.0
C17—C12—C11	118.3 (4)	C14A—C15A—H15A	119.7
C12—C13—C7	122.4 (3)	C16A—C15A—C14A	120.5 (3)
C14—C13—C7	120.0 (4)	C16A—C15A—H15A	119.7
C14—C13—C12	117.5 (3)	C15A—C16A—C18A	122.4 (3)
C13—C14—H14	119.2	C17A—C16A—C15A	117.7 (3)
C13—C14—C15	121.5 (4)	C17A—C16A—C18A	119.9 (3)
C15—C14—H14	119.2	C12A—C17A—H17A	118.7
C14—C15—H15	119.5	C16A—C17A—C12A	122.7 (3)
C16—C15—C14	120.9 (4)	C16A—C17A—H17A	118.7
C16—C15—H15	119.5	C16A—C18A—H18A	108.0
C15—C16—C18	121.8 (4)	C16A—C18A—H18B	108.0
C17—C16—C15	117.8 (4)	C16A—C18A—H18C	108.2
C17—C16—C18	120.4 (4)	C16A—C18A—H18D	108.2
C12—C17—H17	118.7	H18A—C18A—H18B	107.3
C16—C17—C12	122.5 (4)	H18C—C18A—H18D	107.3
C16—C17—H17	118.7	C19A—C18A—C16A	117.1 (4)
C16—C18—H18E	108.6	C19A—C18A—H18A	108.0
C16—C18—H18F	108.6	C19A—C18A—H18B	108.0
H18E—C18—H18F	107.6	C19B—C18A—C16A	116.5 (7)
C19—C18—C16	114.6 (4)	C19B—C18A—H18C	108.2
C19—C18—H18E	108.6	C19B—C18A—H18D	108.2
C19—C18—H18F	108.6	C18A—C19A—H19A	116.4
C18—C19—H19	116.5	C20A—C19A—C18A	127.3 (5)
C20—C19—C18	127.1 (6)	C20A—C19A—H19A	116.4
C20—C19—H19	116.5	C18A—C19B—H19B	115.2
C19—C20—H20E	120.0	C20A—C19B—C18A	129.7 (7)
C19—C20—H20F	120.0	C20A—C19B—H19B	115.2
H20E—C20—H20F	120.0	C19A—C20A—H20A	120.0
C1A—O1A—H1A	114 (3)	C19A—C20A—H20B	120.0
O1A—C1A—C2A	117.7 (3)	C19B—C20A—H20C	120.0
O2A—C1A—O1A	122.7 (3)	C19B—C20A—H20D	120.0
O2A—C1A—C2A	119.6 (3)	H20A—C20A—H20B	120.0
C1A—C2A—C3A	104.9 (3)	H20C—C20A—H20D	120.0
C1A—C2A—C4A	112.4 (3)		
			
O1—C1—C2—C3	−71.8 (4)	O1A—C1A—C2A—C8A	−132.5 (3)
O1—C1—C2—C4	172.0 (3)	O2A—C1A—C2A—C3A	−69.5 (4)
O1—C1—C2—C8	48.6 (4)	O2A—C1A—C2A—C4A	175.0 (3)
O2—C1—C2—C3	104.1 (3)	O2A—C1A—C2A—C8A	50.9 (4)
O2—C1—C2—C4	−12.1 (4)	C1A—C2A—C4A—C5A	−75.7 (4)
O2—C1—C2—C8	−135.5 (3)	C1A—C2A—C8A—C7A	72.0 (4)
C1—C2—C4—C5	−74.3 (3)	C1A—C2A—C8A—C10A	−59.2 (4)
C1—C2—C8—C7	70.0 (4)	C2A—C4A—C5A—C6A	−53.0 (4)
C1—C2—C8—C10	−61.5 (4)	C2A—C8A—C10A—C11A	−161.1 (3)
C2—C4—C5—C6	−54.1 (4)	C3A—C2A—C4A—C5A	169.8 (3)
C2—C8—C10—C11	−159.1 (3)	C3A—C2A—C8A—C7A	−170.6 (3)
C3—C2—C4—C5	171.5 (3)	C3A—C2A—C8A—C10A	58.2 (4)
C3—C2—C8—C7	−172.5 (3)	C4A—C2A—C8A—C7A	−54.0 (4)
C3—C2—C8—C10	56.0 (4)	C4A—C2A—C8A—C10A	174.8 (3)
C4—C2—C8—C7	−54.9 (3)	C4A—C5A—C6A—C7A	55.8 (4)
C4—C2—C8—C10	173.6 (3)	C5A—C6A—C7A—C8A	−56.6 (3)
C4—C5—C6—C7	55.1 (4)	C5A—C6A—C7A—C9A	65.4 (3)
C5—C6—C7—C8	−54.6 (4)	C5A—C6A—C7A—C13A	−177.6 (3)
C5—C6—C7—C9	68.1 (4)	C6A—C7A—C8A—C2A	57.0 (3)
C5—C6—C7—C13	−174.7 (3)	C6A—C7A—C8A—C10A	−169.3 (2)
C6—C7—C8—C2	55.9 (3)	C6A—C7A—C13A—C12A	136.7 (3)
C6—C7—C8—C10	−169.7 (3)	C6A—C7A—C13A—C14A	−48.3 (4)
C6—C7—C13—C12	136.5 (4)	C7A—C8A—C10A—C11A	66.2 (4)
C6—C7—C13—C14	−46.9 (4)	C7A—C13A—C14A—C15A	−175.3 (3)
C7—C8—C10—C11	67.5 (4)	C8A—C2A—C4A—C5A	51.0 (4)
C7—C13—C14—C15	−177.4 (3)	C8A—C7A—C13A—C12A	17.2 (4)
C8—C2—C4—C5	52.6 (4)	C8A—C7A—C13A—C14A	−167.8 (3)
C8—C7—C13—C12	17.6 (4)	C8A—C10A—C11A—C12A	−50.6 (4)
C8—C7—C13—C14	−165.9 (3)	C9A—C7A—C8A—C2A	−62.6 (4)
C8—C10—C11—C12	−51.0 (4)	C9A—C7A—C8A—C10A	71.1 (3)
C9—C7—C8—C2	−64.7 (4)	C9A—C7A—C13A—C12A	−105.1 (3)
C9—C7—C8—C10	69.7 (4)	C9A—C7A—C13A—C14A	69.8 (4)
C9—C7—C13—C12	−104.5 (4)	C10A—C11A—C12A—C13A	21.0 (5)
C9—C7—C13—C14	72.0 (4)	C10A—C11A—C12A—C17A	−159.0 (3)
C10—C11—C12—C13	20.5 (5)	C11A—C12A—C13A—C7A	−4.0 (5)
C10—C11—C12—C17	−160.0 (3)	C11A—C12A—C13A—C14A	−179.1 (3)
C11—C12—C13—C7	−3.5 (5)	C11A—C12A—C17A—C16A	178.5 (3)
C11—C12—C13—C14	179.9 (3)	C12A—C13A—C14A—C15A	−0.1 (5)
C11—C12—C17—C16	−179.0 (4)	C13A—C7A—C8A—C2A	178.5 (3)
C12—C13—C14—C15	−0.7 (5)	C13A—C7A—C8A—C10A	−47.7 (3)
C13—C7—C8—C2	176.8 (3)	C13A—C12A—C17A—C16A	−1.4 (5)
C13—C7—C8—C10	−48.7 (3)	C13A—C14A—C15A—C16A	0.0 (5)
C13—C12—C17—C16	0.6 (6)	C14A—C15A—C16A—C17A	−0.5 (5)
C13—C14—C15—C16	0.1 (6)	C14A—C15A—C16A—C18A	178.8 (4)
C14—C15—C16—C17	0.8 (6)	C15A—C16A—C17A—C12A	1.2 (5)
C14—C15—C16—C18	−176.9 (4)	C15A—C16A—C18A—C19A	20.0 (6)
C15—C16—C17—C12	−1.1 (6)	C15A—C16A—C18A—C19B	−32.9 (7)
C15—C16—C18—C19	−84.1 (6)	C16A—C18A—C19A—C20A	−119.2 (5)
C16—C18—C19—C20	−129.7 (6)	C16A—C18A—C19B—C20A	121.4 (13)
C17—C12—C13—C7	177.0 (3)	C17A—C12A—C13A—C7A	175.9 (3)
C17—C12—C13—C14	0.3 (5)	C17A—C12A—C13A—C14A	0.8 (5)
C17—C16—C18—C19	98.3 (6)	C17A—C16A—C18A—C19A	−160.7 (4)
C18—C16—C17—C12	176.6 (4)	C17A—C16A—C18A—C19B	146.4 (6)
O1A—C1A—C2A—C3A	107.0 (3)	C18A—C16A—C17A—C12A	−178.1 (3)
O1A—C1A—C2A—C4A	−8.5 (4)		

### (1S,4aS,10aR)-1,4a-Dimethyl-7-(prop-2-en-1-yl)-1,2,3,4,4a,9,10,10a-octahydrophenanthrene-1-carboxylic acid Hydrogen-bond geometry (Å, º)

*Cg*3 and *Cg*6 are the centroids of the C12*A*–C17*A* and C12–C17 rings, respectively.

**Table:**

*D*—H···*A*	*D*—H	H···*A*	*D*···*A*	*D*—H···*A*
O1—H1···O1^i^	0.87 (2)	1.88 (3)	2.720 (5)	161 (8)
O2—H2···O2^i^	0.87 (2)	1.82 (3)	2.656 (5)	162 (7)
O1*A*—H1*A*···O2*A*^i^	0.87 (2)	1.78 (2)	2.652 (3)	174 (4)
C10*A*—H10*B*···O2*A*	0.99	2.51	2.917 (4)	104
C10—H10*D*···O1	0.99	2.58	2.969 (4)	104
C4—H4*B*···*Cg*3^ii^	0.99	3.00	3.978 (4)	170
C4*A*—H4*AB*···*Cg*6^iii^	0.99	2.90	3.863 (4)	165

Symmetry codes: (i) −*x*+1, −*y*+1, *z*; (ii) *x*+1/2, −*y*+1/2, −*z*+1; (iii) *x*−1/2, −*y*+1/2, −*z*+1.

## References

[bb1] Adelekan, A. M., Prozesky, E. A., Hussein, A. A., Ureña, L. D., van Rooyen, P. H., Liles, D. C., Meyer, J. M. & Rodríguez, B. (2008). *J. Nat. Prod.* **71**, 1919–1922. 10.1021/np800333r 18855442

[bb2] Ahmad, F., Parveen, M., Alam, M., Azaz, S., Malla, A. M., Alam, M. J., Lee, D. U. & Ahmad, S. (2016). *J. Mol. Struct.* **1116**, 317–332.

[bb3] Bruker (2014). *APEX2, *SAINT* and *XPREP*.* Madison, Wisconsin, USA.

[bb4] Bruno, I. J., Cole, J. C., Kessler, M., Luo, J., Motherwell, W. D. S., Purkis, L. H., Smith, B. R., Taylor, R., Cooper, R. I., Harris, S. E. & Orpen, A. G. (2004). *J. Chem. Inf. Comput. Sci.* **44**, 2133–2144. 10.1021/ci049780b 15554684

[bb5] Butler, M. S. (2004). *J. Nat. Prod.* **67**, 2141–2153. 10.1021/np040106y 15620274

[bb6] Dolomanov, O. V., Bourhis, L. J., Gildea, R. J., Howard, J. A. K. & Puschmann, H. (2009). *J. Appl. Cryst.* **42**, 339–341.

[bb7] Etter, M. C., MacDonald, J. C. & Bernstein, J. (1990). *Acta Cryst.* B**46**, 256–262. 10.1107/s0108768189012929 2344397

[bb8] Green, N. J., Willis, A. C. & Sherburn, M. S. (2016). *Angew. Chem. Int. Ed.* **55**, 9244–9248. 10.1002/anie.201604527 27375221

[bb9] Groom, C. R., Bruno, I. J., Lightfoot, M. P. & Ward, S. C. (2016). *Acta Cryst.* B**72**, 171–179. 10.1107/S2052520616003954 PMC4822653 27048719

[bb10] Hanson, J. R. (2009). *Nat. Prod. Rep.* **26**, 1156–1171. 10.1039/b807311m 19693413

[bb11] Hitchcock, P. B., Avent, A. G., Martsinovich, N., Troshin, P. A. & Taylor, R. (2005). *Org. Lett.* **7**, 1975–1978. 10.1021/ol050441t 15876033

[bb12] Jagtap, M., Ashok, B. K., Chavan, S. S., Chandola, H. M. & Ravi­shankar, B. (2011). *Indian J. Nat. Prod. Res.* **2**, 335–344.

[bb13] Krause, L., Herbst-Irmer, R., Sheldrick, G. M. & Stalke, D. (2015). *J. Appl. Cryst.* **48**, 3–10. 10.1107/S1600576714022985 PMC4453166 26089746

[bb14] Macrae, C. F., Sovago, I., Cottrell, S. J., Galek, P. T. A., McCabe, P., Pidcock, E., Platings, M., Shields, G. P., Stevens, J. S., Towler, M. & Wood, P. A. (2020). *J. Appl. Cryst.* **53**, 226–235. 10.1107/S1600576719014092 PMC6998782 32047413

[bb15] Merritt, A. T. & Ley, S. V. (1992). *Nat. Prod. Rep.* **9**, 243–287. 10.1039/np9920900243 1436738

[bb16] Noumbissie, E. B., Kapnang, H., Fomum, Z. T., Martin, M. T. & Bodo, B. (1992). *J. Nat. Prod.* **55**, 137–139.

[bb17] Parsons, S., Flack, H. D. & Wagner, T. (2013). *Acta Cryst.* B**69**, 249–259. 10.1107/S2052519213010014 PMC3661305 23719469

[bb18] Sheldrick, G. M. (2015*a*). *Acta Cryst.* A**71**, 3–8.

[bb19] Sheldrick, G. M. (2015*b*). *Acta Cryst.* C**71**, 3–8.

[bb20] Simard, F., Legault, J., Lavoie, S. & Pichette, A. (2014). *Phytochemistry*, **100**, 141–149. 10.1016/j.phytochem.2013.12.018 24485585

[bb21] Spek, A. L. (2020). *Acta Cryst.* E**76**, 1–11. 10.1107/S2056989019016244 PMC6944088 31921444

[bb22] Sun, H. D., Huang, S. X. & Han, Q. B. (2006). *Nat. Prod. Rep.* **23**, 673–698. 10.1039/b604174d 17003905

[bb23] Tonga, J. L., Kamdem, M. H. K., Pagna, J. I. M., Fonkui, T. Y., Tata, C. M., Fotsing, M. C. D., Nkengfack, E. A., Mmutlane, E. M. & Ndinteh, D. T. (2022). *Arab. J. Chem.* **15**, 104150, 1–7.

[bb24] Ulubelen, A., Öksüz, S., Kolak, U., Tan, U., Bozok-Johansson, C., Çelik, C., Kohlbau, H. J. & Voelter, W. (1999). *Phytochemistry*, **52**, 1455–1459.

[bb25] Ye, Y., Kim, S. T., Jeong, J., Baik, M. H. & Buchwald, S. L. (2019). *J. Am. Chem. Soc.* **141**, 3901–3909. 10.1021/jacs.8b11838 PMC6402987 30696242

[bb26] Zhu, L. & Chen, L. (2019). *Cell. & Mol. Biol. Lett.* **24**, 1–11.

[bb27] Zhu, Z., Lin, L., Xiao, J. & Shi, Z. (2022). *Angew. Chem. Int. Ed.* **61**, e202113209. 10.1002/anie.202113209 34889493

